# Deciphering the Diagnostic Potential of Small Non-Coding RNAs for the Detection of Pancreatic Ductal Adenocarcinoma Through Liquid Biopsies

**DOI:** 10.3390/ijms26168108

**Published:** 2025-08-21

**Authors:** Hadas Volkov, Rani Shlayem, Noam Shomron

**Affiliations:** 1 Gray Faculty of Medical and Health Sciences, Tel Aviv University, Tel Aviv 69978, Israel; hadasvol@mail.tau.ac.il (H.V.); ranishlayem@mail.tau.ac.il (R.S.); 2 Edmond J. Safra Center for Bioinformatics, Tel Aviv University, Tel Aviv 69978, Israel

**Keywords:** PDAC, ncRNA, miRNA, lncRNA, ML, NGS, bioinformatics

## Abstract

Pancreatic Ductal Adenocarcinoma (PDAC) is one of the most lethal cancers, accounting for a significant proportion of cancer-related deaths globally. Despite advancements in medical science, treatment options for PDAC remain limited, and the prognosis is often poor. Early detection is a critical factor in improving patient outcomes, but current diagnostic methods often fail to detect PDAC until it has advanced to a late stage. In this context, the development of more effective diagnostic tools is of paramount importance. In this study, we explored the potential of non-coding RNAs (ncRNAs) as diagnostic markers for PDAC using cell-free nucleotides and liquid biopsies. Leveraging the power of Next Generation Sequencing (NGS), bioinformatics analysis, and machine learning (ML), we were able to identify unique RNA signatures associated with PDAC. Our findings revealed twenty key genes, including microRNAs (miRNAs), long-non-coding RNAs (lncRNAs), and miscellaneous RNAs that demonstrated high classification accuracy. Specifically, our model achieved a classification accuracy of 87% and an area under the receiver operating characteristic curve (AUC) of 91%. These ncRNAs could potentially serve as robust biomarkers for PDAC, offering a promising avenue for the development of a non-invasive diagnostic test. This could revolutionize PDAC diagnosis, enabling earlier detection and intervention, which is crucial for improving patient outcomes. This work lays the groundwork for future research, with the potential to significantly enhance PDAC diagnosis and therapy.

## 1. Introduction

PDAC is the most common type of pancreatic cancer, accounting for over 90% of all pancreatic neoplasms [1]. Despite advancements in treatment and diagnosis [2], PDAC continues to have a high morbidity rate and poor prognosis. It is the fourth leading cause of cancer-related deaths globally, contributing to 22% of gastrointestinal cancer fatalities and 5% of all cancer deaths [3]. The disease primarily affects individuals over 60, with more than 80% of cases diagnosed in this age group. Known risk factors include smoking and obesity. Additionally, genetics play a significant role in PDAC development, with 5% of all cases carrying a germline mutation in oncogenes like *KRAS* or tumor suppressors such as *CDKN2A* and *TP53* [4].

Potential causes contributing to the high morbidity rate of the disease are the treatment options available for PDAC, which are notably limited and often ineffective [5]. One of the primary challenges in treating PDAC is its inherent resistance to conventional cancer treatments. Chemotherapy, a standard treatment for many types of cancer, often proves ineffective against PDAC. This resistance can be attributed to the dense stromal environment of the tumor, which can create a physical barrier that impedes the delivery of chemotherapeutic agents to the cancer cells [6]. Surgical resection, while potentially curative, is only an option for a small percentage of patients, as most are diagnosed when the disease is already at an advanced stage. Even when surgery is possible, it is a high-risk procedure given the pancreas’s deep location within the abdominal cavity, and it is often associated with significant morbidity and mortality.

The difficulties encountered in PDAC treatment have prompted a shift towards more individualized therapeutic approaches. Personalized medicine, which customizes treatment plans based on the unique attributes of a patient’s malignancy, may offer a fresh path for patient care, but a more critical challenge and a higher reward lies in the early detection of the disease [7]. Traditional diagnostic methods, such as imaging techniques, are often unable to detect PDAC until it has advanced to a late stage [8]. The most commonly used biomarker for PDAC, Cancer Antigen 19-9 (CA19-9), has limitations in sensitivity and specificity, and its levels can be influenced by non-cancerous conditions, leading to false positives. Moreover, not all PDAC patients exhibit elevated CA19-9 signals, limiting its utility as a universal diagnostic marker [9,10,11]. However, the landscape of PDAC diagnosis is evolving with the advent of novel biomarkers, particularly non-coding RNAs [12,13,14,15]. These biomarkers can be detected in bodily fluids such as blood or saliva, offering a less invasive and more accessible testing process. The use of advanced technologies like NGS; Deep Sequencing or high-throughput sequencing, bioinformatics analysis, and ML has enabled the identification of unique RNA signatures associated with PDAC. These RNA signatures could potentially serve as reliable diagnostic markers, enabling early detection and intervention, which is crucial for improving patient outcomes.

The search for indicative biomarkers in liquid biopsies has been extensive, with methods aiming to detect early signs of cancer for general and specific malignancies [12,13,16,17,18] with diverse and potentially exotic non-coding genomic biotypes [19,20]. The ncRNAs are a class of RNA molecules that do not code for proteins but play crucial roles in various biological processes. These cRNAs are broadly categorized by size. Small ncRNAs, typically less than 200 nucleotides in length, include microRNAs (miRNAs), small interfering RNAs (siRNAs), and PIWI-interacting RNAs (piRNAs). Long non-coding RNAs (lncRNAs), which exceed this length, represent another major class. These ncRNAs are involved in gene regulation, both at the transcriptional and post-transcriptional level. For instance, miRNAs bind to messenger RNAs (mRNAs) and inhibit their translation into proteins [21]. Similarly, siRNAs are involved in the RNA interference (RNAi) pathway, leading to targeted degradation of specific mRNAs. RNA subtype piRNAs are primarily found in the germline and are essential for maintaining genome stability. The study of ncRNAs has opened up new avenues for understanding disease mechanisms, including cancer, and they are being explored as potential diagnostic and therapeutic targets. PDAC has been targeted as a specific cancer type due to the shortage of high-impact biomarkers in the early stage [21]. The majority of approaches aim at identifying changes in the regulation of miRNAs associated with tumor progression and proliferation via high-throughput sequencing of known and specific short sequences identified in solid tumor assays and their trace in blood plasma [22,23,24,25]. Even though miRNA-based markers are able to discriminate and classify PDAC patients with relatively high accuracy and specificity, other types of RNAs have also shown high perspective as potential candidates, such as mitochondrial specific RNA [20], lncRNA [26], piRNA, and most recently, circular RNA [27] (circRNA) with accuracy rates of up to 80%.

With the advent of high-throughput NGS assays, which allow the capture of diverse RNA signatures in a single experiment, we developed a comprehensive framework to assess multiple ncRNAs from blood-derived cell-free RNA (cfRNA). This framework allows detailed characterization of PDAC progression and proliferation by leveraging the expansive feature set of the RNA transcriptome. Moreover, it identified significant correlations between patient demographics and treatment regimens, paving the way for more personalized and effective treatment strategies. Taken together, our findings demonstrate that profiling non-coding cell-free RNA (nccfRNA) transcriptome reveals a robust and dynamic RNA signature for the significant diagnosis of PDAC disease.

## 2. Results

### 2.1. Enabling Non-Coding Profiling of the Cell-Free RNA Transcriptome

**Figure 1:**
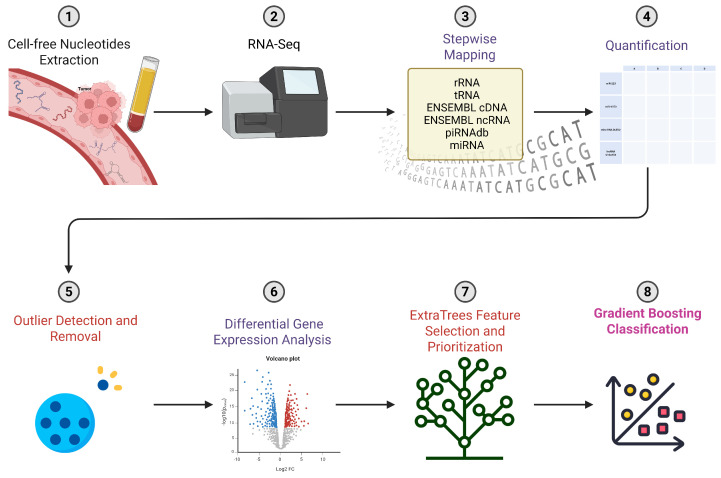
Schematic illustration of the integrated bioinformatics and ML workflow. The process commences with a stepwise alignment procedure, an iterative method ensuring optimal sequence alignment, critical for accurate downstream analysis. Subsequently, the workflow identifies differentially expressed genes, pivotal in deciphering gene expression patterns and their biological significance. The workflow then prioritizes genes using the ExtraTrees algorithm, a robust ML technique that aids in the selection of salient genes for further investigation. The final stage employs gradient boosting classification, a potent ML algorithm, for data classification and predictive modeling.

To quantify cell-free RNA-seq data from human plasma, we developed a step-by-step mapping process to accurately identify and count different types of RNA. This type of alignment procedure is influenced by existing methods but unique to our usage intention of accurately and specifically retrieving signals that are previously known to bear high potential distinction between PDAC and healthy plasma samples. We emphasize the importance of miRNAs as a potential high specificity feature compared to other known RNAs by allowing up to two mismatches during mapping to the miRBase sequence database, compared to full matches only while mapping to ENSEMBL’s non-coding sequences and piRNAdb. We subsequently aggregate read mappings from these diverse sources and quantify expression levels for tens of thousands of gene counts, while excluding specific and high-quality alignments to transfer and ribosomal RNA sequences (Figure 1).

**Figure 2:**
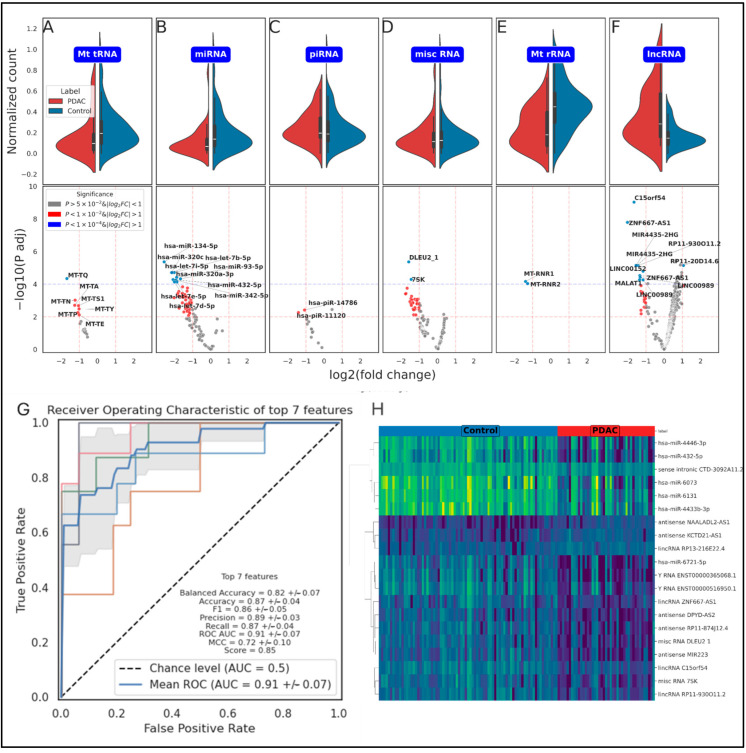
Detailed analysis of ncRNA biotypes and classification performance. A visual representation of expression analysis of various ncRNA biotypes and the performance of the gradient-boosting classification model. (**A**–**F**) Violin plots coupled with partial volcano plots for six distinct ncRNA biotypes: mitochondrial tRNA, miRNA, piRNA, misc RNA, mitochondrial rRNA, and lncRNA. These plots offer a detailed view of the distribution of expression levels for each biotype in both PDAC and control samples. The violin plots illustrate the density and spread of the data, while the volcano plots highlight the differentially expressed genes for each biotype, providing a clear picture of the significant variations between the two groups. (**G**) Presents a receiver operating characteristic (ROC) curve with Cross-Validation (CV), showcasing the performance of the model when classifying based on the top 7 genetic features. The AUCcurve is also indicated, offering a single summary measure of the model’s predictive power. (**H**) a heatmap of the top 20 features, representation of the expression levels of these features in PDAC and control samples. The heatmap uses the Ward metric for hierarchical clustering, which groups samples based on the similarity of their expression patterns for these top features. This figure underscores the effectiveness in distinguishing between PDAC and healthy plasma samples using plasma-derived ncRNAs and highlights the potential of these RNAs as biomarkers for PDAC.

Following this, we perform counts normalization and Differential Gene Expression (DGE) analysis to identify differentially expressed genes between PDAC and control samples. We then employ a robust ExtraTrees algorithm to rank the importance of genetic features and train a gradient-boosted classification model on the top genetic features. This blend of bioinformatics and ML methodologies allows us to achieve exceptional results that outperform existing approaches in classification tasks using plasma-derived ncRNAs. Our top-performing model includes the seven highest-ranking genes selected by the ExtraTrees algorithm, achieving 87% accuracy, 91% area under the receiver operating characteristic curve (AUC) (at its peak), and an F1 score of 86%, which is the weighted harmonic mean of precision and recall (Figure 2G, Supplementary Figure S2). While the combined panel demonstrates high classification accuracy, the predictive power stems from the synergistic combination of these features, as some individual genes show overlapping expression between the two cohorts.

### 2.2. Differential Expression Analysis

**Figure 3:**
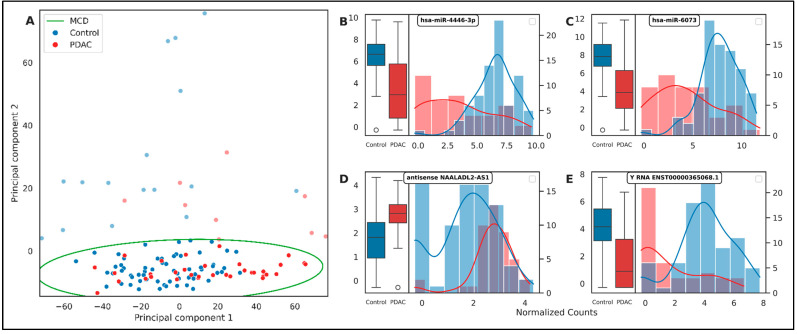
(**A**) PCA of the TMM normalized count matrix. of the first two principal components. The data points are color-coded based on their labels, ‘Control’ and ‘PDAC’. The decision boundaries of the MCD estimator are also shown, identifying potential outliers in the data. The plot provides an overview of the data distribution and the difficulty of separation in an unsupervised manner between the two classes. (**B**–**E**) These subplots present the distribution of normalized counts for four top genes in both ‘Control’ and ‘PDAC’ groups. Each subplot consists of a boxplot (**left**) and a histogram with a kernel density estimate (**right**). The boxplots provide a summary of the central tendency, dispersion, and skewness of the gene expression data, while the histograms offer a visual interpretation of data distribution. The genes are labeled in each subplot. These plots visualize the differences in gene expression between ‘Control’ and ‘PDAC’ groups for the top-most important genes. Note the overlapping distributions for some genes, highlighting the importance of a multi-gene panel for accurate classification.

The initial phase of our DGE analysis involved the application of Principal Component Analysis (PCA). PCA, a statistical procedure that employs an orthogonal transformation to convert a set of observations to a lower dimension, was utilized. Despite its utility, the PCA plot did not exhibit a distinct unsupervised separation between the Control and PDAC groups (Figure 3A). This outcome highlighted the intricate and high-dimensional nature of the gene expression data, necessitating a more robust and flexible method such as supervised gradient boosting. Furthermore, we employed the Minimum Covariance Determinant (MCD) method for outlier detection and removal, with a majority of these outliers originating from the Control group. MCD, a robust estimator of multivariate location and scatter, is particularly effective for outlier detection in high-dimensional datasets. The removal of these outliers aimed to enhance the robustness of our subsequent analyses and ensure that our findings were not disproportionately influenced by these extreme observations.

**Figure 4:**
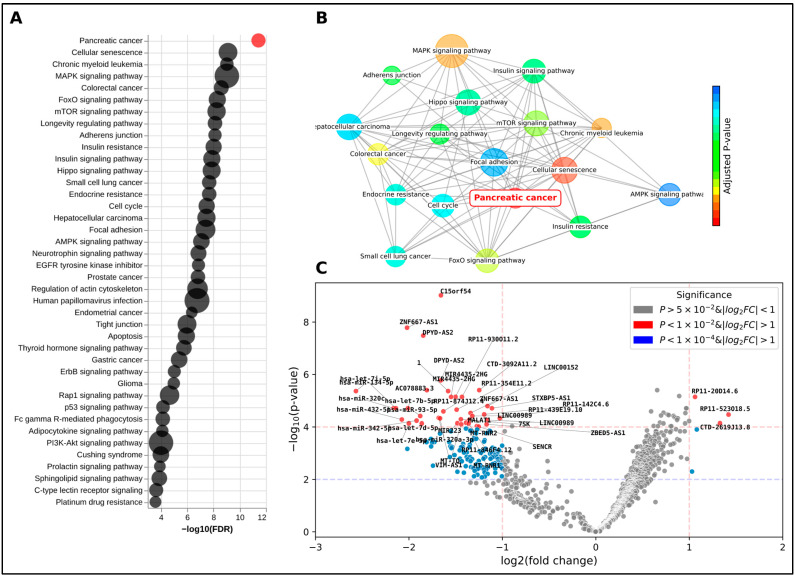
Gene set enrichment and pathway analysis of top 20 genes. Results of the gene set enrichment and pathway analysis of the top 20 genes identified from the DGE analysis. (**A**) Dot plot of enriched terms, with each dot representing a specific term. The size of the dot corresponds to the count of the term, and the color represents the adjusted *p*-value. The pancreatic cancer term is highlighted in red, indicating its significance. (**B**) Network plot of enriched terms, illustrating the relationships between the terms based on their gene overlap. Each node represents a term, and the edges represent the overlap between the terms. The pancreatic cancer term is highlighted in red. (**C**) Volcano plot from the edgeR analysis, providing a visual representation of the differentially expressed genes. Each dot represents a gene, with the *x*-axis showing the log2 fold change and the *y*-axis showing the −log10 adjusted *p*-value.

A comprehensive DGE analysis was conducted with the objective of identifying statistically significant differentially expressed genes between PDAC and control samples (Supplementary Table S3). Following this, a feature selection process was implemented to extract the top-ranking candidates from these genes, as previously outlined. Subsequently, these top 20 genes (Table 1) were subjected to gene set enrichment and pathway analysis. This served as an orthogonal validation method, assessing the genes’ association with pancreatic and cancer-related pathways (Figure 4A). Notably, KEGG’s pancreatic cancer pathway emerged as the top-ranking pathway, with an adjusted *p*-value of 3.75 × 10^−12^, significantly ahead of the subsequent pathways. In addition, several signaling pathways closely associated with cancer, aging, and cell proliferation also ranked highly. Interestingly, pathways related to normal pancreatic function, such as insulin signaling and resistance, were also significantly enriched (Supplementary Table S3), highlighting the broad biological disruption caused by the tumor. For instance, cellular senescence, a state of stable cell cycle arrest linked to aging and cancer, is highly correlated with PDAC, which is predominantly diagnosed in patients in their 70s [28]. The *MAPK* signaling pathway, despite encompassing 1025 genes, is a common pathway in cancer due to its strong association with cell proliferation. It is also important to note that other types of gastrointestinal cancers and malignancies share common risk factors such as age, smoking, and certain genetic predispositions, as well as overlapping ncRNA biomarkers (Supplementary Table S2).

Among the top differentially expressed genes are lncRNAs *C15orf54*, *ZNF667-AS1*, and *DPYD-AS2* (Table 1). These genes have been previously described in the literature as potential prognostic biomarkers for gastrointestinal cancers and PDAC [29]. However, in our ExtraTrees prioritization model, these lncRNA genes did not rank as the top three differentiators. Interestingly, *ZNF667-AS1* did not even appear in the top 20, signaling its limited ability to act as a strong biomarker for PDAC.

### 2.3. Correlation Analysis of Non-Coding Gene Expression and Clinical Features in PDAC Patients

**Figure 5:**
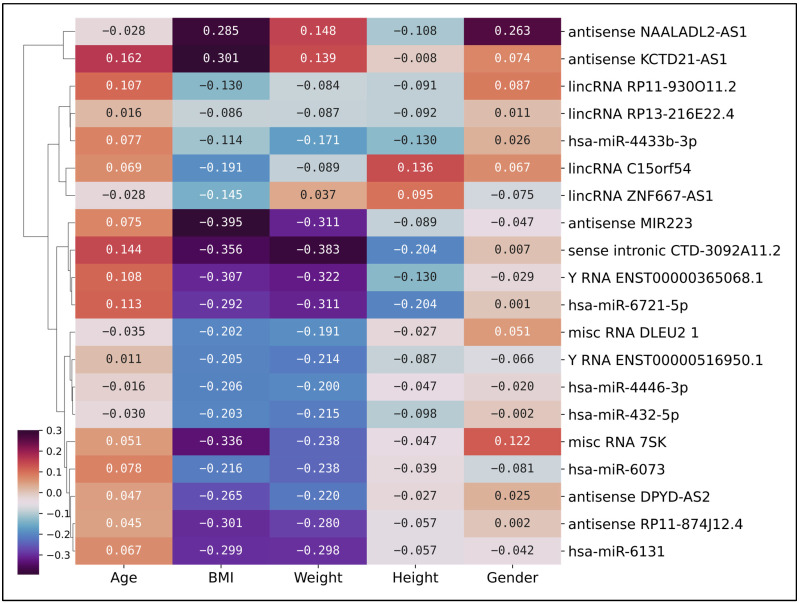
Clustered heatmap of correlation coefficients in the PDAC cohort. A clustered heatmap of correlation coefficients between the normalized read counts of the top 20 non-coding genes and the clinical attributes (age, BMI, weight, height, gender) of the PDAC samples. Each cell in the heatmap represents the correlation coefficient between a specific gene and a clinical feature. The color scale, ranging from dark blue (negative correlation) to light yellow (positive correlation), indicates the strength and direction of the correlation. The dendrogram on the *y*-axis groups genes based on the similarity of their correlation patterns with the clinical features. Notably, BMI and weight display correlations peaking at −0.39 with certain genes, including the lncRNA antisense MIR223. Conversely, gender and height demonstrate correlations approximating zero with the selected genes. The heatmap underscores the intricate interplay between gene expression and clinical features in PDAC.

To investigate potential associations between gene expression and clinical parameters within the disease group, we performed a correlation analysis. This analysis was conducted exclusively on the PDAC patient cohort (*n* = 43), assessing the relationship between the normalized read counts of our top 20 non-coding genes and the clinical attributes of these patients. This analysis was executed to investigate potential associations between gene expression and clinical parameters, which could elucidate the biological underpinnings of PDAC. Figure 5 depicts a clustered heatmap that reveals a spectrum of correlation coefficients between the selected genes and the clinical features. Notably, BMI and weight display correlations peaking at −0.39 with certain genes, indicating a moderate inverse relationship. This inverse correlation suggests that an increase in BMI and weight is associated with a decrease in the expression of these genes, and vice versa. This observation could imply a potential involvement of these genes in metabolic processes associated with PDAC.

Interestingly, the lncRNA *antisense MIR223* gene exhibits a correlation coefficient of −0.39 with PDAC patients. This moderate inverse relationship suggests that as the levels of *antisense MIR223* increase, the incidence or severity of PDAC tends to decrease, and vice versa. The role of *miR-223* in cancer is complex and varies across different types of cancer. It is typically repressed in hepatocellular carcinoma and leukemia, although higher expression levels of *miR-223* are linked to colorectal and recurrent ovarian cancers, and pancreatic cancers [30]. In some cases, *miR-223* downregulation correlates with high tumor burden, disease aggressiveness, and poor patient prognosis [31].

Another gene of interest in our analysis is the small nuclear RNA (snRNA) *7SK*, which exhibits a correlation coefficient of −0.34 with BMI. The 7SK snRNA is a ncRNA consisting of 331–333 base pairs. Previous studies have indicated that overexpression of *7SK* snRNA promotes apoptosis in cancerous cells, suggesting its potential role as an endogenous anti-cancer agent [32]. In our study, the inverse correlation with BMI could imply a potential involvement of *7SK* snRNA in metabolic processes associated with PDAC. However, further research is required to elucidate the precise role of *7SK* snRNA in PDAC and its potential as a therapeutic target.

Conversely, gender and height demonstrate correlations approximating zero with the selected genes, indicating an absence of a linear relationship. Despite the late-onset nature of PDAC, the selected top 20 genes do not exhibit a significant correlation with age. This observation could suggest that the expression of these genes is not directly influenced by aging processes, but rather by other yet unidentified factors or mechanisms. This correlation analysis underscores the intricate interplay between gene expression and clinical features in PDAC, highlighting the necessity for further investigations to fully comprehend these relationships and their implications for PDAC pathogenesis and treatment strategies.

### 2.4. Investigation of Predicted miRNA Target Proteins

The ExtraTrees routine to prioritize top candidate genes for classification has resulted in 20 ncRNA candidates, from which six are miRNAs known to be downregulated in PDAC patients [22,23,33,34,35,36]. Since miRNAs regulate gene expression by binding to target mRNAs, thereby affecting protein levels, we hypothesized that these dysregulated miRNAs might point to key proteins of functional significance. We used NcPath (v1.0) to identify proteins whose corresponding mRNAs are experimentally validated targets of our candidate miRNAs, filtering for strong evidence in the miRTarBase [37] (Supplementary Table S4). The proteins that were found in the miRTarBase are *CDKN1A*, *E2F1*, *E2F2*, *PIK3R1*, *BAK1*, *RAD51*, and *BCL2L1*. These proteins are known to play significant roles in various biological processes and pathways. *CDKN1A*, a cyclin-dependent kinase inhibitor, plays a crucial role in cell cycle regulation and is often found to be dysregulated in various cancers [38]. *E2F1* and *E2F2*, members of the *E2F* family of transcription factors, are involved in cell cycle control and apoptosis, and their aberrant expression is associated with tumorigenesis [39]. *PIK3R1* is a regulatory subunit of the *PI3Ks*, which are part of the *PI3K*/*AKT*/*mTOR* pathway, a critical pathway in cancer that regulates cell survival and growth [40]. *BAK1* and *BCL2L1* are key players in the regulation of apoptosis, a process often disrupted in cancer cells to evade cell death [41]. *RAD51* is involved in the repair of DNA double-strand breaks, and its overexpression has been linked to resistance to radiation therapy in several cancers [42]. The interactions of these proteins with the identified miRNAs could provide valuable insights into the molecular mechanisms underlying PDAC, potentially revealing novel therapeutic targets and diagnostic markers.

## 3. Discussion

PDAC remains a formidable challenge in oncology, with survival rates that have remained stagnant over the past decades [43]. The majority of patients are diagnosed at advanced stages, often with metastases, making them eligible only for palliative care. Even for those with localized tumors, radical surgery often fails to ensure long-term survival, with median survival post-diagnosis ranging from eight to ten months, and most patients experiencing early tumor relapse [44]. Survival rates are influenced by a multitude of factors, including the type and stage of cancer at diagnosis, treatment approach, patient’s age and overall health, lifestyle choices, and variations in healthcare systems.

While survival is predominantly determined by the stage of the tumor, both long-term and short-term survival have been observed in patients diagnosed with early-stage tumors. Recent research has highlighted the prognostic potential of mainly circulating miRNAs profiling in various cancers, but also for other biotypes of ncRNAs. This is due to their altered expression during tumorigenesis and their partial stability in bodily fluids. When examining miRNAs specifically in blood plasma, there are two primary ways in which they can circulate. They can either be encapsulated within exosomes or they can be bound to proteins [45]. While circulating exosomes serve as crucial carriers of tumor-derived miRNAs and present a potential avenue for biomarker discovery.

Our study presents a comprehensive analysis of ncRNAs in the context of PDAC patients, demonstrating their potential as diagnostic markers through liquid biopsies. We leveraged advanced technologies such as NGS, bioinformatics analysis, and ML to identify unique RNA signatures associated with PDAC. The multi-aligner stepwise sequence mapping procedure developed in this study allowed us to accurately retrieve signals from plasma samples. This approach, while influenced by existing methods, was unique in its intention to specifically retrieve signals previously known to bear high potential distinction between PDAC and healthy plasma samples. Notably, the pancreatic cancer pathway emerged as the top-ranking pathway, significantly ahead of subsequent pathways. This finding underscores the potential of these genes as biomarkers for PDAC, specifically in liquid biopsies.

The correlation analysis between the top 20 genes and clinical features of PDAC patients revealed intricate interplays between gene expression and clinical features. For instance, BMI and weight display moderate inverse relationships with certain genes, suggesting potential involvement of these genes in metabolic processes associated with PDAC. Our study also investigated the predicted protein targets of the identified miRNAs. The interactions of these proteins with the identified miRNAs could provide valuable insights into the molecular mechanisms underlying PDAC, potentially revealing novel therapeutic targets and diagnostic markers.

Our study demonstrates that the top seven genes identified through the prioritization process yield a classification with high accuracy and specificity. These results surpass those of comparable methods that focus on specific biotypes or a particular set of RNAs in blood plasma. The top seven genes include three miRNAs: *hsa-miR-4446-3p*, *hsa-miR-6073*, and *hsa-miR-432-5p*. While these miRNAs have been previously linked to various cancers, including PDAC, they are seldom highlighted as top candidates for classification in other published methods. We propose that while other methods prioritize the most differentially expressed miRNAs, this does not necessarily guarantee optimal classification. Our findings underscore the importance of not only considering the most differentially expressed miRNAs but also their potential for effective classification. The top differentially expressed miRNAs may not always be the most informative or relevant for classification tasks. Therefore, a more comprehensive approach, such as the one employed in our study, that considers both differential expression and feature importance, can lead to the identification of more reliable and robust biomarkers for PDAC.

It is important to note that the strength of our diagnostic panel lies in the collective signature of these genes rather than the individual performance of each one. Some of the top-ranking genes, such as *NAALADL2-AS1*, exhibit considerable expression overlap between the PDAC and control groups when viewed in isolation (Figure 3, Supplementary Figure S1). Such markers would perform poorly on their own and would likely lead to a high rate of false positives. However, our multivariate machine learning approach is designed to capture the complex, synergistic interplay between multiple markers. A gene with a seemingly weak individual signal can still provide crucial predictive power when its expression is considered in the context of the other genes in the panel. Therefore, our findings should be interpreted as the discovery of a predictive panel, and the individual components should not be considered strong standalone biomarkers without further validation.

Apart from miRNAs, a Y RNA transcript *ENST00000365068.1* has made the top seven candidates for classification. Y RNAs are a class of ncRNAs that are approximately 100 nucleotides in length. They were first discovered as components of the Ro ribonucleoprotein (RNP) complex in humans. Y RNAs have been implicated in various cellular processes, including DNA replication initiation, regulation of RNA stability, and cellular responses to stress [46]. Recent studies have also suggested a potential role for Y RNAs in cancer [17]. Changes in Y RNA expression have been observed in several types of cancer, and these changes may influence cancer cell proliferation and survival. The design of this study was not intended to elucidate the specific biological role of this Y RNA transcript in PDAC, which remains an area for future research.

Additionally, three lncRNAs complete the group of seven top gene features. One of them, *DLEU2*, is a known prognostic biomarker for PDAC previously described by Qu et al. [47]. Encoded by the *DLEU2* gene, it has been identified as a potential tumor suppressor gene, often deleted in patients with B-cell chronic lymphocytic leukemia. Given its role in tumorigenesis, it is plausible to consider *DLEU2* as a potential classification feature for PDAC. As such, further investigation is required to better assess the role of *DLEU2* as a diagnostic biomarker.

*RP11-874J12.4*, a novel lncRNA [48], has been identified as a significant player in chemoresistance in cancer, as per a study by Liu et al. This lncRNA was found to be overexpressed in gastric cancer tissues and cell lines, and its knockdown increased the sensitivity of these cells to chemotherapeutic drugs. In our study, *RP11-874J12.4* emerged as one of the seven classification features for PDAC, suggesting its potential role in this type of cancer as well. This aligns with the findings of Liu et al., who discovered that *RP11-874J12.4* acts as a sponge for microRNAs, which are typically downregulated in cancer. The role of *RP11-874J12.4* in chemoresistance, as demonstrated in gastric cancer, combined with its significance as a classification feature in our PDAC study, underscores its potential as a key biomarker in cancer diagnosis and treatment. This lncRNA could be pivotal in understanding the mechanisms of chemoresistance in PDAC and could potentially serve as a target for therapeutic intervention.

Lastly, lncRNA *NAALADL2-AS1* has emerged as the top candidate for classification, but there is no evidence in the literature of its role in cancer, in general and in pancreatic cancer specifically. Further examination is required to better assess its role and function in PDAC.

The findings of our study have significant clinical implications for the diagnosis and treatment of PDAC. The identification of unique RNA signatures associated with PDAC, particularly the top 20 genes identified through the ExtraTrees prioritization process, could potentially serve as robust biomarkers for PDAC. Early detection is crucial for improving patient outcomes, as it allows for more treatment options and a better prognosis. Furthermore, our findings could also influence treatment strategies for PDAC. The identified ncRNAs, particularly the lncRNAs and miRNAs, could potentially serve as therapeutic targets. For instance, strategies could be developed to modulate the expression of these ncRNAs to influence the progression of PDAC. Additionally, understanding the role of these ncRNAs in chemoresistance could lead to the development of more effective treatment strategies that can overcome this resistance. However, it is important to note that the translation of these findings into clinical practice would require further validation in larger, independent patient cohorts and potentially in vivo or clinical trials. Nonetheless, our study provides a promising starting point for the development of novel diagnostic and therapeutic strategies for PDAC.

### Limitations

While our study provides important insights into the potential role of ncRNAs as diagnostic markers for PDAC, one of the primary limitations is the relatively small sample size. Our study was based on 122 samples, of which only 43 were PDAC. This small sample size, particularly of PDAC samples, may limit the robustness and generalizability of our findings. Additionally, the samples were collected from two different hospitals. This could introduce variability due to differences in sample collection, processing, and storage procedures between the two institutions. Furthermore, the imbalance in the number of PDAC and control samples could potentially introduce bias. To combat these limitations, we have introduced rigors CV and multiple iterations routines to determine the robustness of the approach while mitigating the potential pitfalls of small-sized datasets. Additionally, the modest size of our PDAC cohort (*n* = 43) precluded more granular subgroup analyses, such as stratifying by age, which would be valuable for exploring age-related effects on gene expression within the disease. Such analyses are fundamental for future validation studies with larger, well-balanced patient cohorts.

Due to low input cfRNA samples, we have opted for a library preparation protocol that is RNA-specific and skips potential loss that might have occurred during additional contamination cleaning steps. Nevertheless, the inclusion of an upfront DNase treatment step during nucleic acid extraction could further reduce competition from cfDNA fragments and potentially increase the yield of target ncRNAs. Additionally, incorporating ribosomal RNA (rRNA) depletion steps, though technically challenging with low quantities, would be highly beneficial. This would reallocate sequencing reads away from abundant rRNA contaminants and significantly increase the detection depth for less abundant but potentially crucial ncRNA species. These optimizations should be considered in the design of future large-scale validation studies.

Finally, the work presented here focuses on the computational multivariate potential of ncRNA ensemble—the essential next step is the experimental validation of our candidate biomarker panel, for instance via qRT-PCR, on the original samples and, more importantly, in a new, larger, and prospectively collected clinical cohort. Such validation is imperative before the clinical translation of this diagnostic signature can be considered.

## 4. Materials and Methods

### 4.1. Cohort Aggregation and Sample Acquisition

A cohort of 122 viable samples was assembled (*n* = 122). This cohort comprised 79 control subjects (*n_control_* = 79) and 43 PDAC patients (*n_PDAC_* = 43), where 5 of the samples were sequenced twice in different stages of the disease (*n_total_* = 122). The sample collection spanned several months, from May 2023 to January 2024, and was conducted across two medical institutions in Israel: (i) Hadassah Medical Center (Jerusalem, Israel). The study protocol was reviewed and approved by their Institutional Review Board (IRB) (Approval number: HMO-0198-14); (ii) Sheba Medical Center (Tel Hashomer, Israel). The study protocol was reviewed and approved by their Institutional Review Board (IRB) (Approval number: SMC-9534-22). Out of the initial 123 samples procured, 1 was excluded due to unsuccessful RNA extraction, which resulted in unreadable outputs. Post collection and plasma isolation, the samples were preserved at −80 °C and dispatched to our laboratory for subsequent processing. Each sample was accompanied by demographic data, including age, BMI and gender (Table 2 and Table S1). Samples from collection through processing were recorded on the Gotsho LIMS system (www.gotsho.com Accessed on 2 August 2024).

Upon examining the distribution of samples within the study, several biases were discernible and posed a challenge for analysis. A significant majority of the samples were procured from Hadassah Medical Center, with Sheba Medical Center contributing no control samples. The control group exhibited a higher female representation, while the patient group was predominantly male. Age disparities were also evident, with the control group averaging nearly 20 years younger than the patient group. Additionally, the control group had a higher proportion of non-smokers. This was later partially mitigated during specification of covariates in the differential gene expression linear model as described below.

### 4.2. Cell-Free Small RNA Isolation from Blood Plasma

For the purification of cell-free nucleic acids from plasma samples, we used the QIAamp Circulating Nucleic Acid Kit (Qiagen, Venlo, The Netherlands) [49], which provides a structured method for this purpose. The process begins with the lysis of plasma samples using proteinase K and Buffer ACL under denaturing conditions to release nucleic acids from their protein or vesicle-bound states. After lysis, Buffer ACB is added to adjust the binding conditions, allowing the nucleic acids to adhere to a silica membrane within the QIAamp Mini columns. The bound nucleic acids are then washed with Buffer ACW1, Buffer ACW2, and ethanol to remove residual contaminants. Finally, the nucleic acids are eluted in Buffer AVE. This method is designed to handle large sample volumes and facilitate the recovery of nucleic acids, making it applicable for the sequencing of small cell-free RNA fragments. The samples’ output was assessed using random sampling using Qubit RNA High Sensitivity (Invitrogen, Carlsbad, CA, USA) [50] and Bioanalyzer RNA Small RNA chip (Agilent, Santa Clara, CA, USA) [51] to ensure minimal loss of material.

### 4.3. Library Preparation for Cell-Free Small RNA-seq

For the purpose of turning the purified cell-free nucleic acids into a library suitable for sequencing, we used the SMARTer smRNA-Seq Kit for Illumina (Takara, Shiga, Japan) [52]. This kit is specifically designed for the preparation of sequencing libraries from small RNA fragments. The process involves polyadenylation of the RNA, followed by cDNA synthesis using template switching technology, which incorporates adapters required for sequencing. After amplification and size selection to ensure the correct library size, the samples are prepared for sequencing. The library preparation was carried out using standardized protocols suitable for the sequencing of small cell-free RNA fragments. The cDNA libraries prepared were assessed with Bioanalyzer DNA High Sensitivity chip (Agilent, Santa Clara, CA, USA) [51], and samples that did not meet minimal criteria were excluded from analysis (1 sample).

### 4.4. RNA-seq Quality Control, Stepwise Alignment, and Quantification

RNA-seq reads were trimmed with fastp [53] (v.0.23.4), quality assessed using FastQC [54] (v.0.12.1), and visualized using MultiQC [55] (v.1.21). Initial contamination filtering of ribosomal and transfer [56] RNAs, as well as coding DNA (ENSEMBL release 86), was performed using Bowtie2 [57] (v.2.4.5), allowing complete matches only. An identical sequential mapping procedure using Bowtie2 was performed to retrieve read mapping to piRNAdb [58] and ENSEMBL’s ncRNA database. Resulting non-mapped reads were mapped to miRBase [59] mature and hairpin sequences (release 22.1) using Bowtie [60]. Quantification of mature miRNA sequences was facilitated using miRTOP [61] (v.0.4.25). Quantification of piRNA and ncRNA was made via manual examination of the alignment files and SAMtools [62] (v.1.19.2) commands. (Supplementary Figures S3 and S4)

### 4.5. Differential Expression Analysis and Count Normalization

Aggregated quantifications were loaded into R and converted to edgeR [63] (v.4.0) objects, where counts were aggregated to the gene level from transcripts. Count filtration was performed using the filterByExpr function under default settings and resulted in 1388 filtered genes. Count normalization was performed with edgeR weighted trimmed mean of the log expression ratios (TMM) [64]. Outlying samples were discarded from the downstream DGE. Discovery of the outlying samples was facilitated using the MCD [65] method—a robust statistical technique used to estimate the parameters of a multivariate dataset. It is particularly useful for detecting outliers in high-dimensional datasets, such as normalized count data. A 20% contamination parameter offered sufficient balance between the number of discarded samples and overall stability.

Subsequently, DGE comparisons were calculated using a linear model in edgeR. To account for potential confounding variables arising from the known demographic and technical differences between our cohorts, we included age, gender, and hospital of origin as covariates in the model. This approach allows for the identification of gene expression changes associated with the disease label while statistically controlling for the influence of these other factors. The full model formula was as follows:

~age + gender + input volume + hospital + label (Control vs. PDAC)(1)

Significant differential expression was considered at adjusted *p* values of <0.01 and Log Fold Change values > 0.5 or <−0.5, resulting in 397 differentially expressed genes.

### 4.6. Feature Importance and Ranking

In the process of our analysis, we leveraged the power of ML to identify the most significant genes that could potentially serve as key features for downstream classification. To achieve this, we utilized the differentially expressed genes and the normalized count matrix as inputs for the ExtraTrees [66] routine, a meta estimator that fits a number of randomized decision trees on various sub-samples of the dataset and uses averaging to improve the predictive accuracy and control over-fitting. The selection of optimal hyperparameters is a critical step in ML algorithms to ensure the best performance. In our study, we employed cross-validation (CV), a robust statistical method that provides an honest assessment of the model’s predictive performance. We chose repeated CV over a single holdout set to provide a more robust performance estimate, a standard practice for datasets of this size where a holdout set would leave insufficient data for model training. To ensure the robustness of our results, we iteratively ran the algorithm 1000 times. In each iteration, the genes were ranked based on their importance. The importance of a gene was determined by the frequency of its appearance in the top 30 features across all iterations. After the completion of the iterative process, we compiled the results and selected the top 20 ranked genes. These genes, which consistently appeared as the most important features across multiple iterations, were then used as the key features for downstream classification tasks. This approach ensures that our model is built on the most informative and relevant features, thereby enhancing its predictive power and reliability.

### 4.7. Gene Set Enrichment and Pathway Analysis

In order to gain a deeper understanding of the biological significance of the top 20 genes identified from the feature importance routine, we conducted a comprehensive gene set enrichment and pathway analysis using NcPath [67] enrichment analysis of human ncRNA and KEGG [68] (release 111.0) signaling pathways. By mapping these genes onto KEGG pathways, we aimed to uncover the key biological pathways that these genes are involved in and to understand their functional context and validate their known role in PDAC-related pathways.

### 4.8. Gradient Boosting Classification

To build our diagnostic model, we used a powerful machine learning technique known as gradient boosting (specifically, the LightGBM (v4.5.0) [69] classifier). The training of the model was conducted using repeated CV. This method is particularly effective in preventing overfitting and providing an honest assessment of the model’s predictive performance. To further enhance the stability and accuracy of our model, we employed a five-fold bagging approach. This technique involves training the model on different subsets of the data and then averaging the predictions. This not only reduces the variance of the model but also helps in avoiding overfitting. The identification of the optimal number of features that best perform classification was achieved using a custom scoring equation. This equation calculates the mean and standard deviation of seven key metrics: balanced accuracy, accuracy, weighted F1 score, precision, and recall. The final score is the average of these seven metrics, providing a comprehensive evaluation of the model’s performance.

## 5. Conclusions

Our study establishes a robust framework for identifying a diagnostic ncRNA signature for PDAC from liquid biopsies, achieving high classification accuracy. The identified multi-gene panel, combining miRNAs and lncRNAs, underscores the value of a comprehensive transcriptomic approach over single-marker analysis. These findings lay the critical groundwork for the development of a non-invasive test for early PDAC detection. The clear next steps involve experimental validation of this signature via qRT-PCR and, most importantly, testing its performance in a large, independent, and well-matched clinical cohort. Successful validation would pave the way for clinical translation, potentially transforming early diagnosis and improving outcomes for this lethal disease

## Figures and Tables

**Table 1:** Summary of top 20 ranked genes identified by ExtraTrees routine. The top 20 genes identified as the most significant features for downstream classification tasks. ‘Mean Importance’ represents the average importance score of each gene across 1000 iterations. ‘Std Importance’ denotes the standard deviation of the importance score, providing an insight into the variability of each gene’s importance. ‘Count Importance’ indicates the number of times each gene appeared in the top 30 features across all iterations, reflecting its consistency in being identified as a significant feature.

	Mean Importance	Std Importance	Count Importance
hsa-miR-4446-3p	0.014894	0.005917	954
hsa-miR-6073	0.014332	0.005727	954
antisense NAALADL2-AS1	0.011694	0.004016	948
Y_RNA ENST00000365068.1	0.012318	0.004933	891
misc RNA DLEU2	0.011427	0.004455	835
antisense RP11-874J12.4	0.011055	0.004207	830
hsa-miR-432-5p	0.010819	0.004178	827
lncRNA C15orf54	0.009861	0.003446	813
hsa-miR-6721-5p	0.011645	0.004544	805
sense intronic CTD-3092A11.2	0.011123	0.004365	801
Y_RNA ENST00000516950.1	0.011071	0.004198	794
antisense DPYD-AS2	0.009608	0.003261	769
hsa-miR-6131	0.011233	0.004398	760
misc RNA 7SK	0.010671	0.004205	752
antisense KCTD21-AS1	0.00875	0.00281	744
lncRNA RP13-216E22.4	0.008391	0.002512	717
antisense MIR223	0.010194	0.003838	712
hsa-miR-4433b-3p	0.010528	0.004083	702
lncRNA RP11-930O11.2	0.009267	0.003152	698

**Table 2:** Summary of demographic and clinical characteristics. Demographic and clinical characteristics of the study cohort, broken down by group (Control and PDAC). The total number of participants (*n* = 122), as well as the number of participants in each group, is provided. For gender, the number and percentage of males and females in each group are given. The mean and standard deviation (SD) are provided for age and Body Mass Index (BMI). The number and percentage of samples collected from each hospital (Hadassah and Sheba) are also included for each group. This summary provides an overview of the cohort’s composition and highlights the differences between the Control and PDAC groups.

	Total (*n* = 122)	Control (*n* = 79)	PDAC (*n* = 43)
Gender			
Male	55 (45.08%)	27 (34.18%)	28 (65.12%)
Female	67 (54.92%)	52 (65.82%)	15 (34.88%)
Age (Mean ± SD)	54.11 ± 16.05	48.60 ± 15.73	64.23 ± 10.98
BMI (Mean ± SD)	25.62 ± 5.00	26.01 ± 5.12	24.76 ± 4.64
Hospital			
Hadassah	110 (90.16%)	79 (100%)	31 (72.09%)
Sheba	12 (9.84%)	0 (0%)	12 (27.91%)

## Data Availability

The data presented in this study are available upon request from the corresponding author. (The data are not publicly available due to privacy or ethical restrictions.)

## Supplementary Materials

The following supporting information can be downloaded at: https://www.mdpi.com/article/10.3390/ijms26168108/s1.
