# Supplementary material for: Deciphering the Diagnostic Potential of Small Non-Coding RNAs for the Detection of Pancreatic Ductal Adenocarcinoma Through Liquid Biopsies
Source: Int J Mol Sci. 2025 Aug 21;26(16):8108. doi: 10.3390/ijms26168108 (PMC12386914; doi:10.3390/ijms26168108)
Supplement: Supplementary file 1 [file ijms-26-08108-s001.zip › SupplementaryMaterial.pdf]

Supplementary Material

Table S1. Patient Demographic and Clinical Information. This table presents the demographic and clinical information of the study cohort. Each row corresponds to a unique patient, identified by a patient code. The table includes information on the patient's gender, age, height (in cm), weight (in kg), and BMI. The date and time of sample collection are also provided. The 'Sequenced' column indicates whether the RNA extraction was unsuccessful (1 = sequenced, 0 = unused). The hospital column indicates the medical institution where the sample was collected. The 'Label' column specifies whether the patient belongs to the 'Control' group or the 'PDAC' group. The full dataset includes both control subjects and pancreatic cancer patients.

Table S2. Top enriched pathways from gene set enrichment analysis. This table presents the top enriched pathways identified from the gene set enrichment analysis of the top 20 genes. The 'Pathway' column lists the name of the pathway as defined in the KEGG database. 'hsaID' represents the unique identifier for each pathway in the KEGG database. 'P-value' and 'P-adjust' columns provide the raw and adjusted P-values respectively, indicating the statistical significance of the enrichment. 'Num Genes' indicates the total number of genes associated with each pathway.

Table S3. Top differentially expressed genes in PDAC samples. Top differentially expressed genes in PDAC samples compared to control samples. The 'logFC' column represents the log2 fold change of gene expression, with negative values indicating downregulation in PDAC samples. 'logCPM' denotes the log counts per million. 'LR' represents the likelihood ratio. 'PValue' and 'FDR' columns provide the raw and adjusted P-values respectively.

Table S4. Summary of miRNA-protein interactions identified from miRTarbase. The table includes the miRNA, target protein, miRTarbase ID, Entrez ID, experimental methods used to validate the interaction, the level of support for the interaction, and the reference. The proteins listed are those identified as top candidates by the ExtraTrees algorithm and are known to be involved in PDAC.

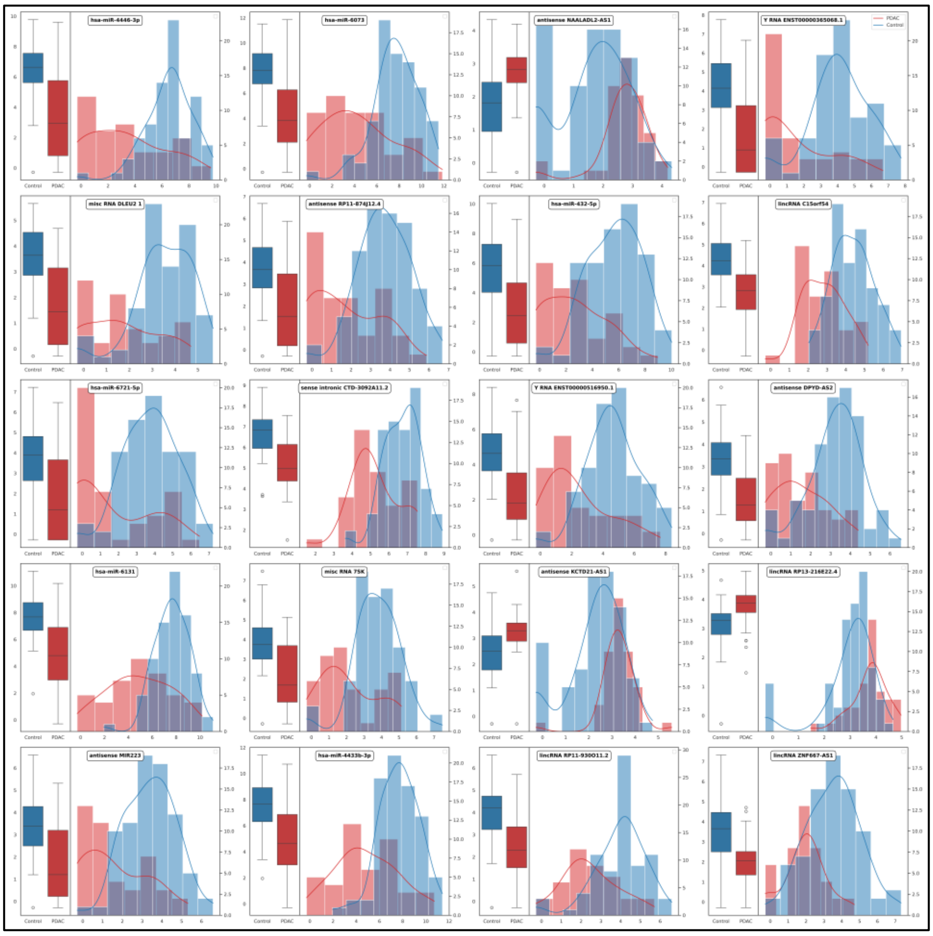

Figure S1. These subplots present the distribution of normalized counts for the 20 top genes in both 'Control' and 'PDAC' groups. Each subplot consists of a boxplot (left) and a histogram with a kernel density estimate (right). The boxplots provide a summary of the central tendency, dispersion, and skewness of the gene expression data, while the histograms offer a visual interpretation of data distribution. The genes are labeled in each subplot. These plots visualize the differences in gene expression between 'Control' and 'PDAC' groups for the top genes.

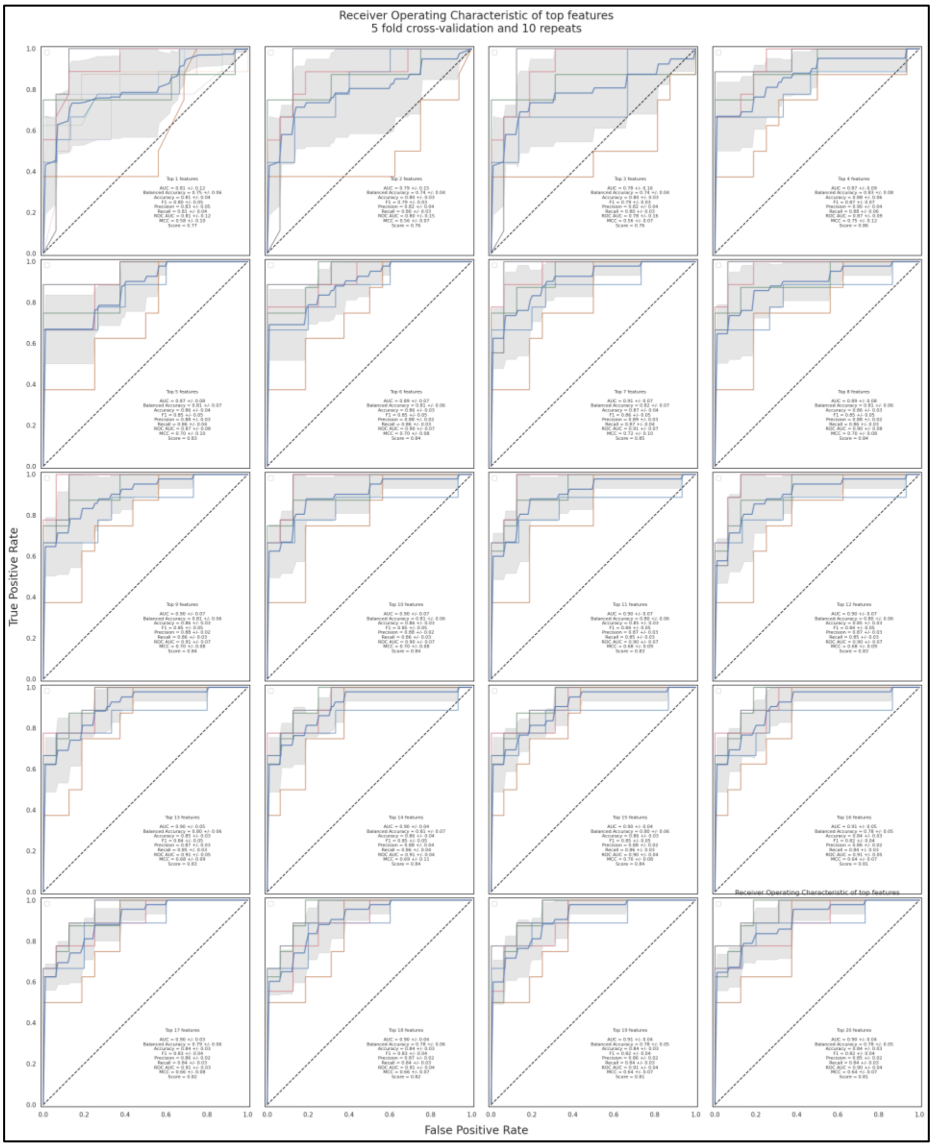

Figure S2. This figure presents a Receiver Operating Characteristic (ROC) curve with CV, showcasing the performance of the model when classifying the top 20 genetic features. The AUC is also indicated, offering a single summary measure of the model's predictive power. The score metric also included and reveals that the top 7 genes are providing optimal classification.

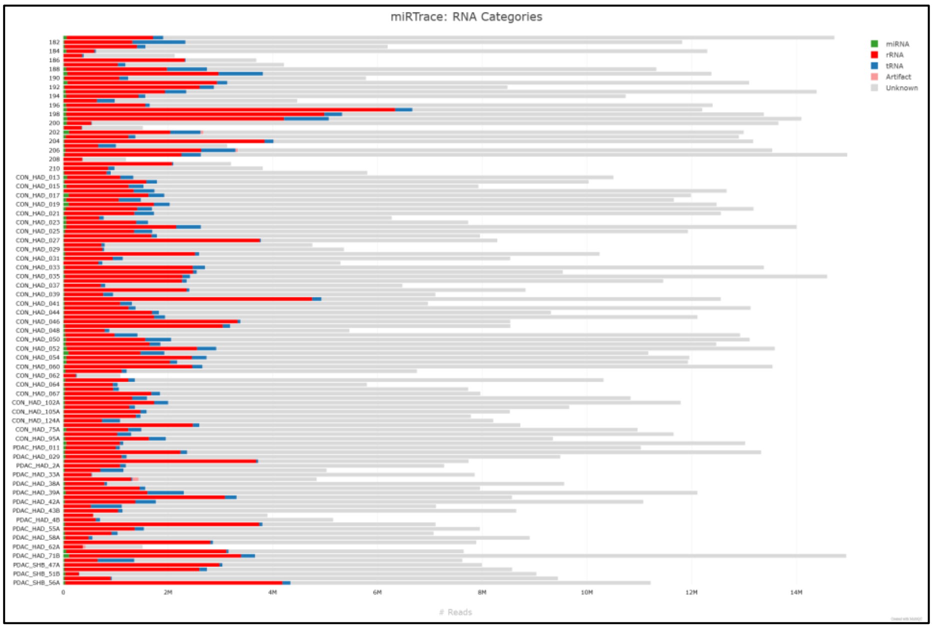

Figure S3. Distribution of RNA Categories in the miRTrace Analysis. The figure illustrates the proportion of different RNA categories identified in the miRTrace analysis, including miRNA, rRNA, tRNA, Artifacts, and Others. The distribution provides insights into the composition of the RNA sample and highlights the prevalence of specific RNA types.

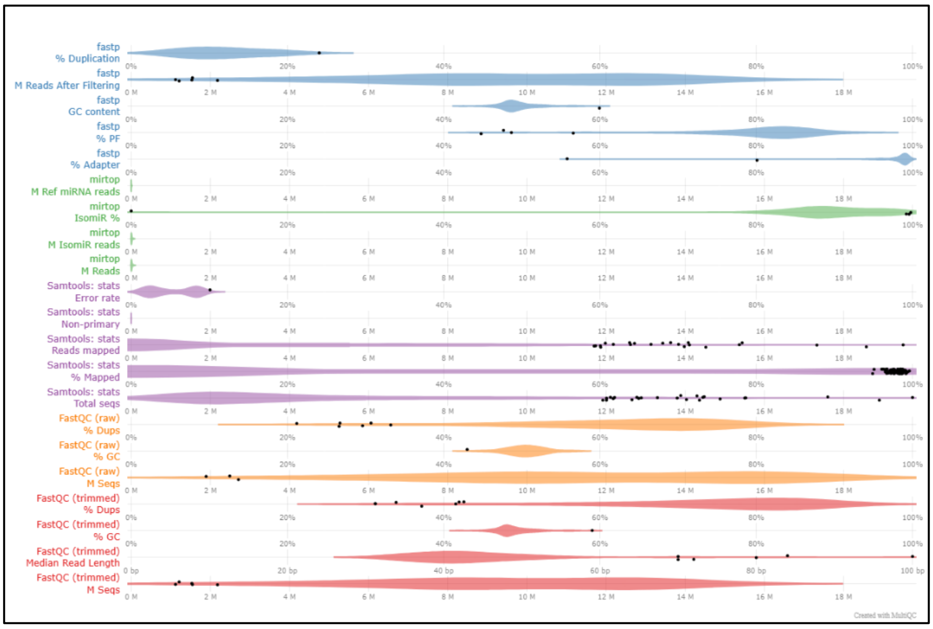

Figure S4. Overview of Raw Sequencing Data Metrics. This figure presents a comprehensive summary of the raw sequencing data generated in this study. It includes various metrics such as the total number of reads, read length distribution, quality scores, GC content, and others. These metrics provide valuable insights into the quality and characteristics of the sequencing data, serving as a foundation for subsequent bioinformatics analyses.
